# Prognostic Value of Tumor-Infiltrating Lymphocytes in Resected Pancreatic Ductal Adenocarcinoma: A Single-Center Retrospective Australian Study

**DOI:** 10.7759/cureus.103580

**Published:** 2026-02-14

**Authors:** Harine Siribaddana, Prasad Jayaratne, Sarushen Gounden, Daniel Whittaker, Matthew Burge, Kayla Tran, Jai W Hoff, Chin Li Tee, Manju D Chandrasegaram

**Affiliations:** 1 General Surgery, The Prince Charles Hospital, Brisbane, AUS; 2 Anatomical Pathology, The Prince Charles Hospital, Brisbane, AUS; 3 Medical Oncology, The Prince Charles Hospital, Brisbane, AUS; 4 General Surgery, Redcliffe Hospital, Redcliffe, AUS

**Keywords:** australia, hematoxylin and eosin, pancreatic cancer resection, pancreatic ductal adenocarcinoma, prognostic markers, survival analysis, tumor-infiltrating lymphocytes, tumor microenvironment

## Abstract

Introduction

Pancreatic ductal adenocarcinoma (PDAC) has a poor prognosis, even after curative-intent resection. Tumor-infiltrating lymphocytes (TILs) assessed by immunohistochemistry have prognostic value, but standardized assessment on routine H&E sections is not established, particularly in Australian cohorts. This pilot study evaluated stromal TIL density in resected PDAC on H&E sections using the International Immuno-Oncology Biomarker Working Group (ITWG) guidelines and explored its association with survival and key clinicopathological metrics.

Methods

We conducted a retrospective cohort study of 21 consecutive patients who underwent curative-intent resection for histologically confirmed PDAC at an Australian tertiary center between September 2016 and January 2025. Stromal TIL density was quantified on H&E sections and categorized a priori as low (<15%) or high (≥15%) lymphocytic infiltration of the tumor stroma. Overall survival (OS) and disease-free survival (DFS) were analyzed using Kaplan-Meier methods and log-rank tests. Univariable Cox regression was performed, followed by an exploratory multivariable Cox model incorporating stromal TIL category, lymph node ratio (LNR), and vascular invasion.

Results

Median follow-up was 17 months (range, 3-99), with 11 deaths (52%) and 12 recurrences (57%). Using the prespecified threshold of 15% stromal TIL density, 10 patients had low stromal TILs, and 11 had high stromal TILs. Compared with low TILs, high TILs were associated with improved OS (median not reached vs. 16 months; log-rank p = 0.029) and DFS (median not reached vs. nine months; log-rank p = 0.026). In the exploratory multivariable model, high TILs remained associated with improved OS (HR 0.15, p = 0.033) and DFS (HR 0.18, p = 0.024). A higher LNR was associated with poorer OS (HR 3.42 per 10% increase, p = 0.005) and DFS (HR 2.41 per 10% increase, p = 0.010). When analyzed as a continuous variable (per 5% increase), stromal TIL density showed a consistent direction of effect without statistical significance for OS (HR 0.94, p = 0.155) or DFS (HR 0.94, p = 0.115).

Conclusions

In this single-center Australian pilot cohort, high stromal TIL density assessed on routine H&E sections was associated with improved OS and DFS following PDAC resection. These findings support the feasibility and potential prognostic utility of stromal TIL assessment using ITWG guidelines and warrant validation in larger multicenter studies.

## Introduction

The incidence of pancreatic cancer in Australia is rising. In 2025, an estimated 4,825 people were expected to be diagnosed nationally, with this number projected to increase to 6,374 by 2034 (age-standardized rate of 18.3 per 100,000) [1,2]. Despite a modest improvement in five-year relative survival for pancreatic cancer (all stages combined) from 3.1% (1987-1991) to 14% (2017-2021), pancreatic cancer was the fourth leading cause of cancer-related death in Australia in 2023 [1,2]. Pancreatic ductal adenocarcinoma (PDAC) is the predominant histological subtype, accounting for approximately 90% of pancreatic cancers [3].

Surgical resection with perioperative systemic chemotherapy remains the standard of care for resectable PDAC, yet prognosis remains poor even after curative-intent surgery [3]. Population-based cohorts have reported a median overall survival (OS) of approximately 18-25 months after resection [4-6]. In a nationwide cohort with pathological reexamination, the observed 10-year OS was 5.3% after resection [7]. In selected trial populations, adjuvant chemotherapy improved outcomes. Randomized controlled trials have reported a median OS of 53.5 months with modified FOLFIRINOX, 28-31.6 months with gemcitabine plus capecitabine, and 25.5-35.5 months with gemcitabine alone [8-10]. This persistently poor prognosis underscores the need for accessible prognostic biomarkers to stratify risk, guide adjuvant treatment decisions, and identify patients who may benefit from emerging immunomodulatory strategies.

The tumor microenvironment in PDAC is characterized by dense desmoplastic stroma rich in cancer-associated fibroblasts and extracellular matrix [11-13]. This fosters an immunosuppressive niche with a relative paucity of cytotoxic T-cell infiltration and enrichment of suppressive myeloid and regulatory T-cell populations, contributing to immune evasion and treatment resistance [11-13]. Tumor-infiltrating lymphocyte (TIL) assessment has demonstrated prognostic value across multiple solid tumors, with higher TIL density associated with improved survival in triple-negative and HER2-positive breast cancer, melanoma, and colorectal cancer [14-16]. Therefore, TIL assessment may help explain immune-mediated heterogeneity in PDAC outcomes, including long-term survivor phenotypes described in translational studies [17].

A 2020 systematic review and meta-analysis reported that higher TIL density and favorable lymphocyte subsets are associated with improved outcomes after resection for PDAC [18]. However, all 19 included studies relied on immunohistochemistry (IHC), which requires specialized staining and limits routine clinical applicability [18]. The International Immuno-Oncology Biomarker Working Group (ITWG) provides a standardized approach for assessing stromal TILs on routine H&E sections [19]. Despite this, few studies have applied this framework to PDAC, and none have evaluated its prognostic value in an Australian cohort.

To address this gap, we conducted a retrospective single-center pilot study of consecutive patients undergoing curative-intent resection for PDAC at an Australian tertiary center. The primary objective was to quantify stromal TIL density on routine H&E sections using ITWG guidance and to explore its association with OS and disease-free survival (DFS). Secondary objectives were to evaluate associations between stromal TIL density and established clinicopathological markers and to describe the feasibility of applying ITWG-aligned stromal TIL scoring within a standard Australian pathology workflow.

This research was presented as an oral presentation at the Australian and Aotearoa New Zealand Hepatic Pancreatic & Biliary Association and General Surgeons Australia Annual Scientific Meeting (Perth, Australia) on October 17, 2025.

## Materials and methods

Study design

This retrospective cohort study included consecutive patients who underwent curative-intent surgical resection for histologically confirmed PDAC at a single Australian tertiary hospital between September 2016 and January 2025. Patients were excluded if final surgical pathology demonstrated a non-PDAC diagnosis, including non-ductal pancreatic neoplasms or periampullary primaries. Histological variants of PDAC (such as adenosquamous or colloid carcinoma) were excluded to maintain a more homogeneous cohort. The censor date for survival analyses was August 8, 2025. This study was approved by The Prince Charles Hospital Human Research Ethics Committee and is reported in accordance with the STrengthening the Reporting of OBservational studies in Epidemiology (STROBE) guidelines.

Data were collected from electronic medical records and paper charts. Baseline characteristics included age, sex, BMI, Eastern Cooperative Oncology Group performance status, American Society of Anesthesiologists physical status, and carbohydrate antigen 19-9 (CA19-9) levels. Treatment variables included neoadjuvant and adjuvant chemotherapy regimens and serial CA19-9 measurements. Given the small cohort and limited number of events, systemic treatment details were summarized descriptively.

Pathological assessment

Tumors were staged and graded according to the American Joint Committee on Cancer (AJCC), 8th Edition [20]. Resection margins were classified as R0 if the minimum distance from invasive carcinoma to any inked margin was ≥1 mm and R1 if the distance was <1 mm, consistent with contemporary PDAC margin definitions [21]. Lymph node ratio (LNR) was calculated as the ratio of involved to examined lymph nodes and reported as a percentage [22,23]. Histologic regression following neoadjuvant treatment was evaluated using the modified Ryan tumor regression grade (TRG) system [24].

Stromal TILs were assessed on routine H&E sections using ITWG guidelines [19]. TILs were recorded as the percentage of tumor stromal area within the invasive tumor borders occupied by mononuclear inflammatory cells (predominantly lymphocytes and plasma cells), reported in 5% increments [19]. The invasive tumor borders were identified at low magnification, and stromal TILs were then estimated as a single overall percentage representing the average proportion of mononuclear inflammatory cells across the evaluable invasive tumor stroma, rather than a hotspot-only assessment. Higher magnification was used as needed to confirm cell morphology. Areas of necrosis, crush artifact, regressive hyalinization, and prior biopsy sites were excluded from evaluation. In patients treated with neoadjuvant therapy, scoring was restricted to residual viable tumor stroma and excluded acellular treatment-related fibrosis [25]. Scoring was performed by a senior pathology registrar and a consultant pathologist through consensus review.

Because validated TIL thresholds for PDAC have not been established, patients were stratified a priori into low (<15%) and high (≥15%) stromal TILs using a pragmatic threshold informed by TIL literature in other solid tumors [14,16,26]. This categorical approach facilitated clinically interpretable risk stratification and survival analyses. No patients were excluded due to inadequate tissue for TIL assessment. As a sensitivity analysis, TIL density was also modeled as a continuous variable (scaled per 5% increase).

Outcomes

The primary outcome was OS, defined as the time from surgery to death from any cause or censoring. The secondary outcome was DFS, defined as the time from surgery to first documented recurrence on cross-sectional imaging or censoring. Secondary exploratory analyses assessed associations between stromal TIL category and clinicopathological variables, including R0 margin status and LNR.

Statistical analysis

Continuous variables are presented as mean (SD) or median (range), and categorical variables as frequencies (percentages). One patient had a missing preoperative CA19-9 value and was excluded only from analyses involving CA19-9. Between-group comparisons used Mann-Whitney U tests for continuous variables and Fisher’s exact test for categorical variables.

OS and DFS were estimated using Kaplan-Meier (KM) methods and compared using log-rank tests. HRs were computed using univariable Cox proportional hazards regression to explore associations between clinicopathological variables and survival outcomes. Given the limited number of events, an exploratory multivariable Cox regression model was fitted with a restricted number of covariates to minimize overfitting and improve model stability. The model included the stromal TIL category, LNR (per 10% increase), and vascular invasion. Other nodal variables (including N stage and lymphatic invasion) were excluded to avoid collinearity with LNR. Because margin status was associated with the TIL category in this cohort, it was not included to support model stability. Multivariable results were interpreted as hypothesis-generating. Analyses were performed using RStudio (Version 2025.09; Posit Software, PBC, Boston, MA, USA).

## Results

Patient characteristics

The cohort included 21 patients who underwent curative-intent resection for PDAC at a single Australian tertiary center. Of these, 10 patients (48%) had low stromal TILs (<15%), and 11 patients (52%) had high stromal TILs (≥15%). Baseline characteristics were generally similar between the two TIL groups (Table 1). The mean age at surgery was 67.2 years (SD 10.5), and 11 patients (52%) were male. The mean BMI was 28.5 kg/m² (SD 5.4). Preoperative CA19-9 levels were available for 20 patients, of whom eight (40%) had elevated values (>37 U/mL).

**Table 1:** Baseline characteristics of patients who underwent curative-intent resection for PDAC, grouped by stromal TIL category ^*^ CA19-9 was available for 20 patients (one missing); percentages are based on available cases. Percentages are calculated within TIL groups unless stated otherwise. p-Values were calculated using the Mann-Whitney U test for continuous variables and Fisher’s exact test for categorical variables. ASA, American Society of Anesthesiologists physical status; CA19-9, carbohydrate antigen 19-9; ECOG, Eastern Cooperative Oncology Group performance status; PDAC, pancreatic ductal adenocarcinoma; TIL, tumor-infiltrating lymphocyte

Characteristic		Low TILs (n = 10)	High TILs (n = 11)	p-Value	Total (n = 21)
Age at surgery (years), mean (SD)		64.9 (11.5)	69.3 (9.5)	0.675	67.2 (10.5)
Sex, n (%)	Male	6 (60)	5 (45)	0.667	11 (52)
	Female	4 (40)	6 (55)		10 (48)
ECOG, n (%)	1	9 (90)	9 (82)	1.000	18 (86)
	2	1 (10)	2 (18)		3 (14)
ASA, n (%)	1 or 2	6 (60)	3 (27)	0.198	9 (43)
	3 or 4	4 (40)	8 (73)		12 (57)
BMI (kg/m²), mean (SD)		28.1 (6.1)	28.9 (5.2)	0.426	28.5 (5.4)
Preoperative CA19-9 (U/mL), median (range)^*^		22 (5-970)	37 (5-2100)	0.295	29 (5-2100)
Abnormal preoperative CA19-9 (>37 U/mL), n (%)^*^		3 (33)	5 (46)	0.670	8 (40)

Pathological characteristics

Surgical and pathological features are summarized in Table 2. Pancreaticoduodenectomy was performed in 15 patients (71%), corresponding to tumors located in the pancreatic head. The mean tumor size on histology was 32.1 mm (SD 16.7). R0 resection (≥1 mm margin clearance) was achieved in eight patients (38%). Pathological staging revealed six patients (29%) with T3 disease and six patients (29%) with N2 disease. No tumors were classified as T4, and nodal involvement did not exceed N2. Five tumors (24%) were grade 3 (poorly differentiated). Extranodal extension was observed in nine patients (43%), and perineural invasion was present in 20 patients (95%). No patients had metastatic disease at the time of resection. The median LNR was 9% (range 0-30).

**Table 2:** Surgical and pathological characteristics of resected PDAC, grouped by stromal TIL category ^*^ R0 margin status: ≥1 mm clearance from the tumor to the inked margin ^†^ Staging and classification defined by the American Joint Committee on Cancer, 8th Edition p-Values were calculated using the t-test for tumor size, the Mann-Whitney U test for LNR, and Fisher’s exact test for categorical variables. LNR, lymph node ratio; PDAC, pancreatic ductal adenocarcinoma; TIL, tumor-infiltrating lymphocyte

Characteristic		Low TILs (n = 10)	High TILs (n = 11)	p-Value	Total (n = 21)
Location, n (%)	Head	6 (60)	9 (82)	0.361	15 (71)
	Body, neck, and tail	4 (40)	2 (18)		6 (29)
Type of resection, n (%)	Pancreaticoduodenectomy	6 (60)	9 (82)	0.361	15 (71)
	Distal pancreatectomy	4 (40)	2 (18)		6 (29)
Size (mm), mean (SD)		29.2 (11.9)	34.7 (20.3)	0.463	32.1 (16.7)
R0 margin status^*^, n (%)		1 (10)	7 (64)	0.024	8 (38)
LNR (%), median (range)		9 (0-26)	12 (0-30)	0.858	9 (0-30)
Grade^†^, n (%)	1-2	7 (70)	9 (82)	0.635	16 (76)
	3	3 (30)	2 (18)		5 (24)
Vascular invasion present, n (%)		7 (70)	3 (27)	0.086	10 (48)
Lymphatic invasion present, n (%)		4 (40)	2 (18)	0.361	6 (29)
Perineural invasion present, n (%)		10 (100)	10 (91)	1.000	20 (95)
T stage^†^, n (%)	1-2	8 (80)	7 (64)	0.635	15 (71)
	3	2 (20)	4 (36)		6 (29)
N stage^†^, n (%)	0-1	9 (90)	6 (54)	0.149	15 (71)
	2	1 (10)	5 (46)		6 (29)
Extranodal extension present, n (%)		4 (40)	5 (45)	1.000	9 (43)
Stromal TILs (%), median (range)		5 (5-10)	20 (15-35)	-	15 (5-35)

Median stromal TIL density was 15% (range 5-35%). R0 resection was more frequent in the high TIL group (7/11, 64%) than in the low TIL group (1/10, 10%) (p = 0.024). Other pathological features showed no clear differences between TIL groups.

Chemotherapy characteristics

Chemotherapy details are summarized in Table 3. Ten patients (48%) received neoadjuvant chemotherapy, with similar proportions in the low and high TIL groups. The median number of neoadjuvant cycles was 4.5 (range 3-12). Median CA19-9 levels decreased from 140 U/mL (range 28-1600) before neoadjuvant therapy to 34.5 U/mL (range 5-970) after therapy, although this did not reach statistical significance on paired Wilcoxon signed-rank testing (p = 0.064). The Ryan TRG was 1-2 in seven of 10 patients (70%) and grade 3 in three of 10 (30%), with no grade 0 regressions observed. Fifteen patients (71%) received adjuvant chemotherapy, with similar proportions between TIL groups. The median number of adjuvant cycles was 5 (range 1-12).

**Table 3:** Neoadjuvant and adjuvant chemotherapy details of patients who underwent curative-intent resection for PDAC, grouped by stromal TIL category ^*^ Modified Ryan TRG: 0 (complete response); 1 (near-complete response); 2 (partial response); 3 (poor/no response) Percentages are within TIL groups unless stated otherwise. In the Total column, “Received neoadjuvant” and “Received adjuvant” use n = 21; all other rows use the relevant subgroup denominator (neoadjuvant n = 10, adjuvant n = 15). p-Values (comparing low vs. high TILs): Mann-Whitney U test for continuous variables; Fisher’s exact test for categorical variables. Pre- and post-neoadjuvant CA19-9 levels are descriptive. CA19-9, carbohydrate antigen 19-9; PDAC, pancreatic ductal adenocarcinoma; TIL, tumor-infiltrating lymphocyte; TRG, tumor regression grade

Characteristic		Low TILs (n = 10)	High TILs (n = 11)	p-Value	Total
Neoadjuvant chemotherapy (n = 10)					
Received neoadjuvant chemotherapy, n (%)		5 (50)	5 (45)	1.000	10 (48)
Total cycles, median (range)		4 (3-12)	6 (3-10)	0.750	4.5 (3-12)
Modified Ryan TRG^*^, n (%)	1-2	3 (60)	4 (80)	1.000	7 (70)
	3	2 (40)	1 (20)		3 (30)
CA19-9 (U/mL), median (range)	Pre-neoadjuvant	100 (28-1600)	180 (46-420)	-	140 (28-1600)
	Post-neoadjuvant	30 (5-970)	39 (18-72)		34.5 (5-970)
Adjuvant chemotherapy (n = 15)					
Received adjuvant chemotherapy, n (%)		7 (70)	8 (73)	1.000	15 (71)
Total cycles, median (range)		3 (1-12)	5 (3-6)	0.767	5 (1-12)

Survival outcomes

**Figure 1:**
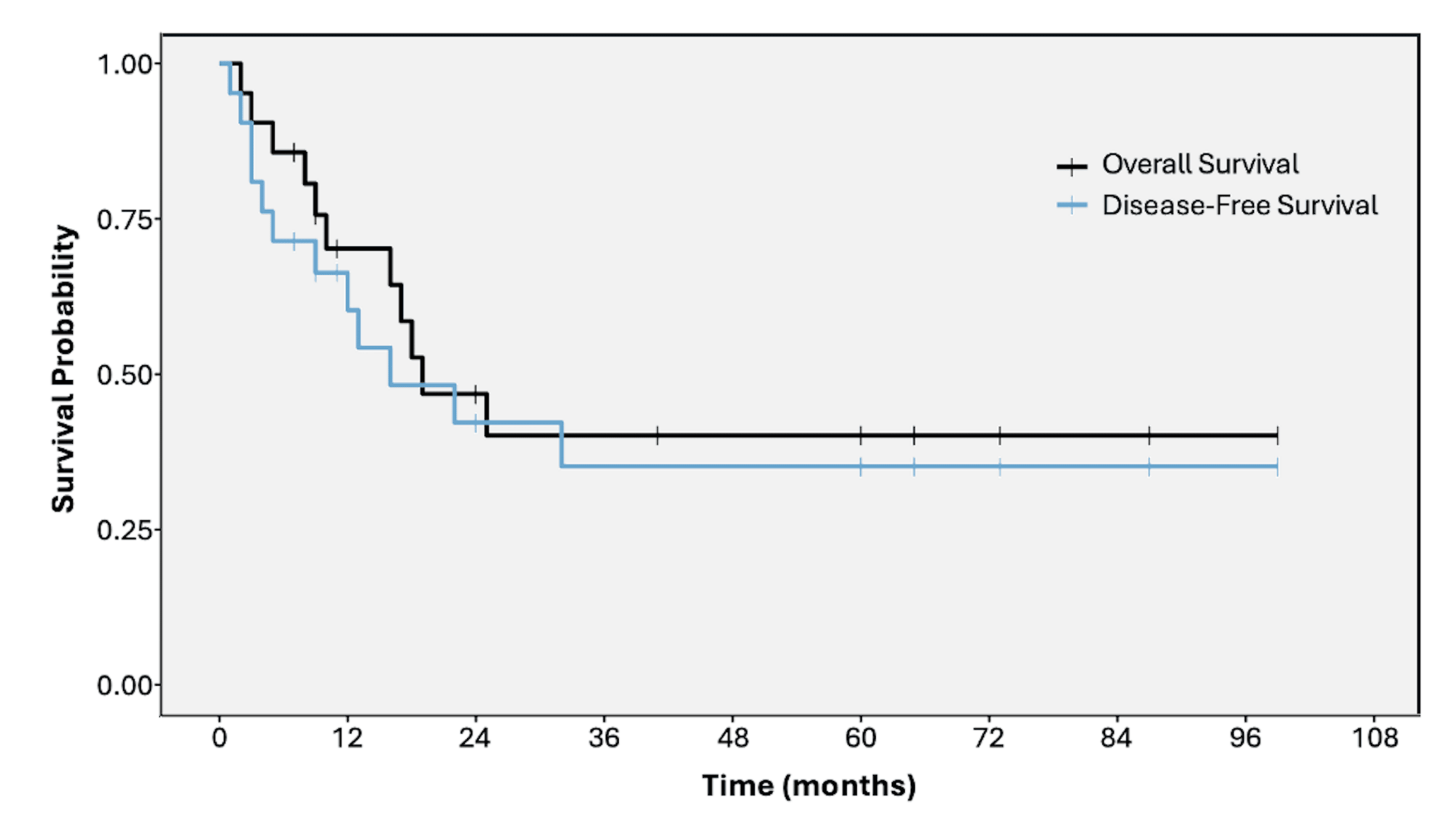
KM curves for OS and DFS in the overall cohort of 21 patients who underwent curative-intent resection for PDAC KM estimates of OS (black) and DFS (blue) from the date of surgery are shown. Crosses indicate censored observations. Median OS was 19 months, and median DFS was 16 months. Follow-up was censored on August 8, 2025. DFS, disease-free survival; KM, Kaplan-Meier; OS, overall survival; PDAC, pancreatic ductal adenocarcinoma

Median follow-up was 17 months (range 3-99). By the censor date, 11 deaths (52%) and 12 recurrences (57%) had occurred. KM analyses for the overall cohort (Figure 1) estimated a median OS of 19 months and a median DFS of 16 months. Among patients alive at censoring (n = 10), median follow-up was 51 months. Among patients without recurrence at censoring (n = 9), median follow-up was 60 months. No perioperative deaths occurred within 30 days of surgery.

**Figure 2:**
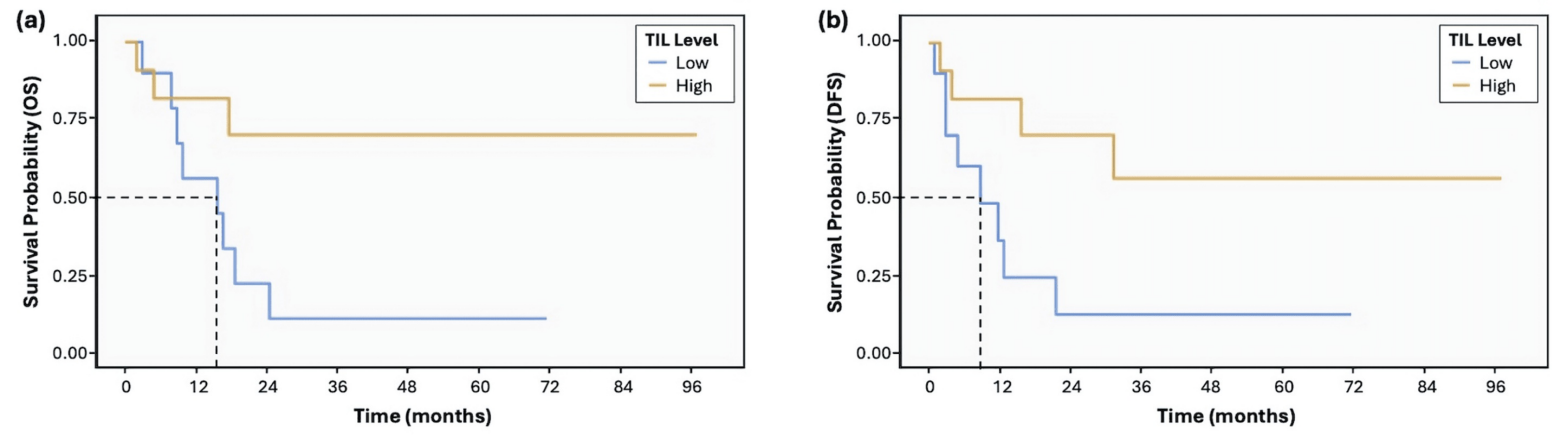
KM curves for (a) OS and (b) DFS stratified by stromal TIL category in resected PDAC (n = 21) Survival from the date of surgery is shown for low stromal TILs (<15%, n = 10) versus high stromal TILs (≥15%, n = 11). Median OS was 16 months in the low TIL group and was not reached in the high TIL group (log-rank p = 0.029). Median DFS was nine months in the low TIL group and was not reached in the high TIL group (log-rank p = 0.026). Dashed lines indicate the median survival estimate for the low TIL group in each panel. Follow-up was censored on August 8, 2025. DFS, disease-free survival; KM, Kaplan-Meier; OS, overall survival; PDAC, pancreatic ductal adenocarcinoma; TIL, tumor-infiltrating lymphocyte

KM analyses demonstrated improved survival among patients with high TILs compared to those with low TILs (Figure 2). Median OS was 16 months in the low TIL group, whereas median OS was not reached in the high TIL group during follow-up (log-rank p = 0.029). Median DFS was nine months in the low TIL group and was not reached in the high TIL group (log-rank p = 0.026).

In sensitivity analyses, modeling stromal TIL density as a continuous variable (per 5% increase) yielded directionally consistent associations, but these did not reach statistical significance. For OS, the HR was 0.94 (95% CI 0.87-1.02; p = 0.155). For DFS, the HR was 0.94 (95% CI 0.87-1.01; p = 0.115).

Regression analysis

Univariable Cox proportional hazards models are presented in Table 4. High stromal TILs were associated with improved OS (HR 0.25, 95% CI 0.07-0.96; p = 0.043) and DFS (HR 0.27, 95% CI 0.08-0.91; p = 0.035). Adverse pathological features associated with poorer OS included higher LNR (HR 2.28 per 10% increase; p = 0.003), grade 3 differentiation (HR 4.30; p = 0.022), vascular invasion (HR 6.40; p = 0.019), lymphatic invasion (HR 4.83; p = 0.010), and N2 disease (HR 8.36; p = 0.044). DFS showed broadly similar associations (Table 4).

**Table 4:** Univariable Cox regression analysis of factors associated with OS and DFS after PDAC resection (n = 21) ^*^ CA19-9 analyses used available cases (n = 20). All other models used n = 21. ^†^ Location “other” includes body, neck, and tail. ^‡^ R0 margin status: ≥1 mm clearance from the tumor to the inked margin. ^§^ As defined by the American Joint Committee on Cancer, 8th Edition. ^¶^ NE, not estimable for OS due to sample size and near-universal presence (n = 20/21). ASA, American Society of Anesthesiologists physical status; CA19-9, carbohydrate antigen 19-9; DFS, disease-free survival; ECOG, Eastern Cooperative Oncology Group performance status; LNR, lymph node ratio; OS, overall survival; PDAC, pancreatic ductal adenocarcinoma; TIL, tumor-infiltrating lymphocyte

Variable	OS HR (95% CI)	p-Value	DFS HR (95% CI)	p-Value
Age (per year)	0.99 (0.93-1.06)	0.832	0.99 (0.92-1.05)	0.655
Sex (female)	0.54 (0.16-1.87)	0.333	0.48 (0.14-1.59)	0.230
ECOG (2 vs. 1)	0.38 (0.05-3.01)	0.362	0.77 (0.16-3.54)	0.734
ASA (3-4 vs. 1-2)	1.59 (0.46-5.48)	0.459	1.80 (0.54-6.06)	0.340
BMI (per kg/m²)	0.95 (0.84-1.07)	0.375	0.96 (0.86-1.07)	0.453
Abnormal preoperative CA19-9 (>37 U/mL)^*^	0.89 (0.25-3.15)	0.852	1.13 (0.34-3.77)	0.837
Location (head vs. other)^†^	1.05 (0.28-3.98)	0.942	1.14 (0.31-4.24)	0.843
Size (per mm)	1.00 (0.97-1.03)	0.854	1.00 (0.97-1.03)	0.854
R0 Margin status^‡^	0.55 (0.15-2.10)	0.384	0.64 (0.19-2.13)	0.463
LNR (per 10% increase)	2.28 (1.34-3.90)	0.003	1.84 (1.14-2.95)	0.012
Vascular invasion present	6.40 (1.35-29.43)	0.019	5.47 (1.44-20.73)	0.012
Lymphatic invasion present	4.83 (1.45-16.07)	0.010	4.77 (1.44-15.77)	0.010
Perineural invasion present	NE^¶^	-	0.99 (0.13-7.81)	0.993
T stage^§^ (3 vs. 1-2)	2.91 (0.62-13.63)	0.176	1.84 (0.49-6.87)	0.365
N stage^§^ (2 vs. 0-1)	8.36 (1.06-66.00)	0.044	4.66 (1.00-21.75)	0.050
Grade^§^ (3 vs. 1-2)	4.30 (1.23-15.01)	0.022	4.76 (1.37-16.57)	0.014
Extranodal extension present	2.21 (0.66-7.42)	0.199	1.81 (0.57-5.76)	0.314
High TILs (≥15% vs. <15%)	0.25 (0.07-0.96)	0.043	0.27 (0.08-0.91)	0.035
Neoadjuvant chemotherapy	0.82 (0.25-2.70)	0.743	1.01 (0.32-3.15)	0.988
Adjuvant chemotherapy	1.43 (0.37-5.51)	0.605	1.57 (0.42-5.94)	0.506

In the exploratory multivariable Cox model (Table 5), which included stromal TIL category, LNR (per 10% increase), and vascular invasion, high stromal TILs remained associated with improved OS (HR 0.15, 95% CI 0.03-0.86; p = 0.033) and DFS (HR 0.18, 95% CI 0.04-0.80; p = 0.024). Higher LNR remained independently associated with poorer outcomes for OS (HR 3.42, 95% CI 1.46-8.05; p = 0.005) and DFS (HR 2.41, 95% CI 1.24-4.68; p = 0.010). The C-index was 0.859 for OS and 0.840 for DFS. Schoenfeld residual testing did not show evidence of proportional hazards violations (global and individual p-values > 0.05). Confidence intervals were wide, reflecting the small cohort and limited number of events; the adjusted estimates should be interpreted as hypothesis-generating.

**Table 5:** Multivariable Cox regression for OS and DFS after curative-intent resection for PDAC (n = 21) Model covariates were prespecified due to the limited number of events. LNR was modeled per a 10 percentage-point increase. Reference categories were low TILs (<15%) and the absence of vascular invasion. DFS, disease-free survival; LNR, lymph node ratio; OS, overall survival; PDAC, pancreatic ductal adenocarcinoma; TIL, tumor-infiltrating lymphocyte

Variable	OS adjusted HR (95% CI)	p-Value	DFS adjusted HR (95% CI)	p-Value
High TILs (≥15% vs. <15%)	0.15 (0.03-0.86)	0.033	0.18 (0.04-0.80)	0.024
LNR (per 10% increase)	3.42 (1.46-8.05)	0.005	2.41 (1.24-4.68)	0.010
Vascular invasion present	4.54 (0.74-27.84)	0.102	3.66 (0.88-15.18)	0.073

## Discussion

In this retrospective cohort study of 21 patients who underwent curative-intent resection for PDAC at a single Australian tertiary center, high stromal TIL density (≥15%) was associated with improved OS and DFS. Survival differences were observed in KM analysis (log-rank p = 0.029 for OS and p = 0.026 for DFS). The association persisted in an exploratory multivariable Cox model adjusted for LNR and vascular invasion, although confidence intervals were wide due to the small number of events. Given that OS analyses were based on 11 deaths and the adjusted model included three covariates, the multivariable estimates should be interpreted cautiously. In this model, a higher LNR was independently associated with poorer outcomes for both OS and DFS (p = 0.005 and p = 0.010, respectively). The persistence of high TILs as a favorable marker after adjustment suggests that host anti-tumor immune response may represent a distinct prognostic axis not fully captured by nodal burden or vascular invasion, potentially identifying an immune-enriched PDAC phenotype, although residual confounding remains possible.

These findings align with existing evidence that immune infiltration is prognostically favorable in resected PDAC. A 2020 systematic review and meta-analysis of 19 studies reported that higher TIL densities and specific favorable lymphocyte subsets (including CD3+, CD4+, and CD8+ lymphocytes) were associated with improved OS [18]. However, all included studies relied on IHC, which requires specialized staining and is less readily scalable for routine reporting. In contrast, our study demonstrates the feasibility of applying ITWG guidelines to stromal TIL scoring on routine H&E sections in PDAC, supporting a pragmatic and low-cost approach that may be more easily incorporated into standard pathology workflows [19].

PDAC is characterized by a dense desmoplastic stroma and an immunosuppressive microenvironment with limited effective cytotoxic infiltration, contributing to immune evasion and treatment resistance [11-13]. Within this setting, higher stromal TIL density may identify an immune-enriched subset with a more favorable phenotype, although immune subsets were not characterized in this study [13,17,27,28].

The associations between stromal TIL category and other pathological features require cautious interpretation. High TILs were more common among patients who achieved an R0 resection (p = 0.024). Given the limited statistical power, this should be interpreted as an association rather than evidence of causality. This pattern may reflect shared underlying tumor biology, differences in local growth patterns, or unmeasured patient and treatment factors, rather than a direct effect of lymphocyte infiltration on technical resectability. Importantly, because margin status was strongly associated with stromal TIL category, it may act as a confounder, and the observed survival association may partially reflect tumor features that also support R0 achievement. In parallel, LNR emerged as a strong adverse prognostic marker and remained associated with poorer outcomes in the adjusted model, consistent with prior studies supporting LNR as a clinically useful nodal metric [22,23]. We did not observe clear differences in stromal TIL density by neoadjuvant treatment exposure, but interpretation is limited by small numbers and the recognized capacity of systemic therapy to alter tumor microenvironment composition and immune infiltration patterns [3,9,12,13,25].

Despite its pilot design, this study has several strengths. The cohort was consecutive with complete follow-up to the censor date, supporting capture of real-world practice across the study period and limiting selection bias. Pathology variables were derived using established frameworks (including AJCC staging and tumor regression grading), and stromal TIL scoring adhered to standardized ITWG guidance on routine H&E sections with consensus assessment by a consultant pathologist and senior registrar [19,20]. Using an H&E-based, ITWG-aligned approach is also cost-neutral and readily scalable within routine pathology workflows compared with immune profiling strategies that require additional IHC, supporting potential generalizability and translational relevance. Finally, the survival signal for high TILs was directionally consistent across KM analysis, univariable Cox modeling, exploratory multivariable modeling, and continuous sensitivity modeling, supporting the internal consistency of the observed associations despite sample size constraints.

Important limitations of this study should be acknowledged. The small sample size (n = 21) from a single institution, with limited events (11 deaths and 12 recurrences), resulted in wide confidence intervals and limited statistical power. The exploratory multivariable estimates should be interpreted cautiously given the low event count and risk of overfitting. Residual confounding is also possible, particularly because margin status was associated with stromal TIL category and was not included in the adjusted model to support stability. Treatment exposure and surveillance intensity were heterogeneous, with temporal changes in systemic therapy across the study period and possible concurrent evolution in perioperative pathways. Systemic treatment heterogeneity could not be comprehensively adjusted for, as event numbers were insufficient to support additional covariates. The prespecified 15% TIL threshold requires external validation, and dichotomization may reduce information compared with continuous modeling. Molecular subtype, genomic alterations, and tumor microenvironment proteomic features were not captured and may be particularly relevant to immune infiltration and prognosis [29]. Although ITWG guidance was followed, scoring on H&E sections does not distinguish immune subsets with divergent prognostic implications [18,30]. Given the retrospective workflow, magnification or block-level sampling strategy was not standardized or routinely recorded. Finally, interobserver assessment was performed by consensus without formal reliability statistics. Nonetheless, the study demonstrates the feasibility of standardized stromal TIL assessment on routine H&E sections in PDAC and provides preliminary effect estimates to inform multicenter validation.

Future studies should prioritize larger multicenter cohorts with prespecified analysis plans to validate the prognostic value of stromal TILs in PDAC using ITWG guidelines. Clinically actionable thresholds specific to PDAC require definition and validation with adequate event counts to ensure robust multivariable modeling. If reproducible across centers, the incremental value of stromal TIL scoring beyond established prognostic markers, including margin status and nodal metrics, should be evaluated, including whether incorporation into synoptic reporting templates improves risk stratification in a clinically meaningful way. Future studies should also quantify interobserver agreement using reliability statistics. Mechanistic studies can evaluate determinants of stromal TIL density in PDAC, including molecular subtype and tumor microenvironment features, and explore whether stromal TILs modify responses to neoadjuvant or adjuvant therapy [12,27,28]. Finally, identifying immune-enriched subgroups using scalable histopathological measures, supported by targeted immunophenotyping in validation cohorts, may refine patient selection for emerging immunotherapies and vaccine strategies [27,28].

## Conclusions

Higher stromal TIL density was associated with improved OS and DFS after curative-intent resection for PDAC in this single-center Australian pilot cohort and was also linked to higher rates of R0 resection. Stromal TIL scoring on routine H&E sections using ITWG guidance was feasible within a standard pathology workflow and represents a scalable, low-cost approach to capturing immune contexture. Given the small sample size and limited number of events, these results should be interpreted as hypothesis-generating, and adjusted estimates should not be considered definitive. Larger prospective multicenter studies are needed to validate this association, refine PDAC-specific TIL thresholds, quantify interobserver reproducibility, and determine whether routine TIL reporting provides incremental prognostic information beyond established clinicopathological factors, such as margin status and nodal metrics.
